# Mechanical Work as an Indirect Measure of Subjective Costs Influencing Human Movement

**DOI:** 10.1371/journal.pone.0031143

**Published:** 2012-02-24

**Authors:** Karl E. Zelik, Arthur D. Kuo

**Affiliations:** 1 Department of Mechanical Engineering, University of Michigan, Ann Arbor, Michigan, United States of America; 2 Department of Biomedical Engineering, University of Michigan, Ann Arbor, Michigan, United States of America; University of Liverpool, United Kingdom

## Abstract

To descend a flight of stairs, would you rather walk or fall? Falling seems to have some obvious disadvantages such as the risk of pain or injury. But the preferred strategy of walking also entails a cost for the use of active muscles to perform negative work. The amount and distribution of work a person chooses to perform may, therefore, reflect a subjective valuation of the trade-offs between active muscle effort and other costs, such as pain. Here we use a simple jump landing experiment to quantify the work humans prefer to perform to dissipate the energy of landing. We found that healthy normal subjects (*N* = 8) preferred a strategy that involved performing 37% more negative work than minimally necessary (*P*<0.001) across a range of landing heights. This then required additional positive work to return to standing rest posture, highlighting the cost of this preference. Subjects were also able to modulate the amount of landing work, and its distribution between active and passive tissues. When instructed to land softly, they performed 76% more work than necessary (*P*<0.001), with a higher proportion from active muscles (89% vs. 84%, *P*<0.001). Stiff-legged landings, performed by one subject for demonstration, exhibited close to the minimum of work, with more of it performed passively through soft tissue deformations (at least 30% in stiff landings vs. 16% preferred). During jump landings, humans appear not to minimize muscle work, but instead choose to perform a consistent amount of extra work, presumably to avoid other subjective costs. The degree to which work is not minimized may indirectly quantify the relative valuation of costs that are otherwise difficult to measure.

## Introduction

Humans appear to value economy of movement [1]–[7], leading to the expectation that the muscles will not usually perform more mechanical work than necessary to complete a motor task. While this general observation seems applicable to costly locomotor tasks such as walking, economy of work may also be relevant to other tasks, such as those primarily involving energy dissipation. In these cases, factors other than work and energy expenditure also clearly influence the preferred movement strategy. For example, humans usually prefer to walk down a long flight of stairs, when it might require less muscular effort simply to fall down them, allowing the work to be performed passively, through soft tissue deformations. Falling might save the energy expended to perform active negative work, but perhaps at the expense of other costs, such as pain or risk of injury. It is difficult to quantify other unknown factors such as pain. But a person's own valuation of their relative costs may be indicated behaviorally by how he or she chooses to perform a task, for example actively vs. passively. The amount and distribution of negative work humans choose to perform may therefore indicate a trade-off between work and other, less easily quantified costs, which may explain why some tasks are performed uneconomically.

A task particularly suited for this inquiry is landing from a jump. Landing collisions dissipate the kinetic energy gained from the descent, largely through negative work performed actively by lower extremity muscles [8]–[11]. The work not due to active muscle is presumably performed passively [12]–[15], through the deformation of soft tissues such as the heel pad [16]–[19], viscera [20]–[22], and vertebral discs [23]. The proportion of work performed actively vs. passively can be modulated, for example, humans can perform “softer” landings, involving greater flexion of the knees, less passive work, and more muscle work [14]. Greater amounts of active negative work might reduce concentrated strain energy that could cause injury to soft tissues, but such landings may also be more metabolically costly.

Alternatively, humans can perform “stiffer” landings, which are more economical from a mechanical work perspective. After all, even a small amount of joint flexion during jump landing could be considered uneconomical, because it entails doing more than the minimum amount of negative work, which may also require subsequent positive work to compensate. One need only practice a few stiff landings to surmise that pain and discomfort are among the countervailing costs. These and other subjective costs almost certainly play a role in many movement strategies, but they are difficult to quantify and compare against each other. It is here that biomechanical measures may be helpful, because they facilitate quantification of the opportunity cost—in terms of work or energy—of a person's preferred movement strategy. In terms of jump landing, a preference to perform more than the minimum amount of work may indicate the relative weighting of other costs on the preferred movement strategy.

The purpose of the present study was to quantify the preferred jump landing strategy of humans in the context of work-like costs. We hypothesized that the preferred landing strategy is a compromise between different costs for both stiffer and softer landings. While stiffer landings may reduce active work, they may entail other costs such as pain or discomfort, perhaps related to excessive passive work performed by soft tissue deformations. Softer landings may reduce passive deformations, but at the cost of increased work overall. Therefore, we tested whether the preferred strategy entails performing more negative work than necessary, and investigated how this work is distributed between active and passive tissues.

## Methods

To test our hypothesis, we measured the work performed by healthy human subjects when landing from jumps. We compared that work against the minimum necessary to land and return to the same final posture, and tested whether the preferred strategy entailed excess work. We also estimated the contributions of active muscles from joint work computed from standard inverse dynamics, and the work of soft tissue deformations based on the total mechanical work performed on the entire body. We measured landings from 8 healthy adult subjects (77.5±14.4 kg, 0.94±0.05 m leg length, 6 male and 2 female) performing vertical jumping over a range of heights.

### Ethics Statement

This study was approved by the University of Michigan Institutional Review Board. All subjects gave written informed consent prior to the experiment.

**Figure 1:**
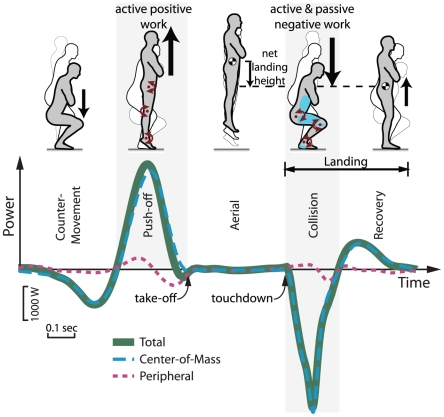
Total mechanical power vs. time for vertical jumping and landing. Subjects jumped vertically, landed and returned to rest. Representative trial demonstrates phases of a jump: Counter-Movement, Push-off, Aerial, Collision and Recovery, defined by zero-crossings of center-of-mass (COM) power. Landing is represented by the Collision and Recovery phases. Total power was estimated as the sum of COM power (due to motion of the COM) and Peripheral power (due to motion relative to the COM). Net landing height was defined as the displacement between maximum aerial height of the COM and standing rest posture.

Subjects performed jumping trials with two landing strategies, treated as separate conditions. In the Preferred condition, subjects were given no landing instructions, whereas in the Soft condition, they were instructed to land as quietly as possible. To avoid affecting subjective preferences, the Soft condition was always tested after Preferred. As a demonstration, one subject also performed an additional condition, Stiff landing, in which he landed flatfooted with his knees fully extended. A trial consisted of standing at rest with one foot on each force plate and with arms crossed, jumping vertically, landing back on the same force plates, and finally returning to the original rest posture (Fig. 1). We defined *net landing height* as the difference between the maximum height of the body center of mass (COM) and the final standing rest posture (Fig. 1). We defined the theoretical minimum amount of work necessary for landing as the gravitational potential energy associated with this displacement. This assumes a person could land with all of the joints vertically aligned so that no joint rotations would occur in landing.

Subjects performed forty jumping trials for each condition, over a range of heights. Subjects received verbal instructions to jump 10 times at each of four different subjective heights between “very low” and “high,” to yield a range of landing displacements. Subjects kept their arms crossed throughout the duration of each trial in order to avoid work done by the arms. A trial was performed again if the subject's feet did not land back on their original force plates.

Ground reaction forces and full-body kinematics were collected according to standard biomechanical procedures for inverse dynamics analysis. Forces were recorded under each foot independently using two in-ground force plates (Advanced Mechanical Technologies Inc., Newton, MA, USA) at 1000Hz. Kinematic data were collected at 100Hz *via* an eight-camera infrared motion capture system (Vicon Motion Systems, Los Angeles, CA, USA) and software (Vicon Nexus v1.5.0). Passive, reflective markers were placed bilaterally on the ankle (lateral and medial malleoli), knee (lateral and medial femoral epicondyles) and hip (greater trochanter). Additionally, we placed four segmental markers on each thigh and shank, and on the pelvis (left/right anterior and posterior superior iliac spines). Three additional markers were placed on each foot (calcaneous, first and fifth metatarsals). Upper-body markers were placed on the neck (at the level of C7), the shoulders (acromion) and the elbows (olecranon bursa). Joint locations for the ankle, knee and hip were computed based on a functional joint center algorithm [24] in commercial software (Visual3D, C-Motion, Germantown, MD, USA). Prior to analysis, forces were filtered at 25 Hz and marker positions at 6 Hz using a 4^th^ order low-pass Butterworth filter.

We estimated 3-D mechanical work for different net landing heights and conditions and the distribution of work between active and passive tissues. We defined Total Mechanical power as that due to motion of the body center-of-mass (COM work rate) plus that due to motion relative to the body COM (Peripheral power). We computed COM work rate based on the dot product of ground reaction force with COM velocity, also derived from forces [25], [26]. We estimated Peripheral power as the time derivative of changes in translational and rotational energy relative to the COM, assuming rigid-body segments. This is also sometimes referred to as “internal work,” (e.g., [27]). As another quantification, we defined Summed Joint power (or simply Joint power) as the combined power from the ankle, knee and hip of both limbs plus the power due to rotation of the trunk about the lumbosacral joint, all using standard rigid-body inverse dynamics methods. We used the Total mechanical work performed on the body, but not captured by rigid-body Joint work estimates as an indicator of soft tissue deformations (similar to [12], [13]). We estimated the Soft Tissue power contribution as the difference between Total Mechanical power and Summed Joint power (Fig. 3). This assumes that most of the soft tissue deformations were captured by COM work rate, which captures both rigid and soft bodies, as opposed to the Peripheral Power contribution, which only quantifies rigid-body motions (see further details in Text S1).

**Figure 2:**
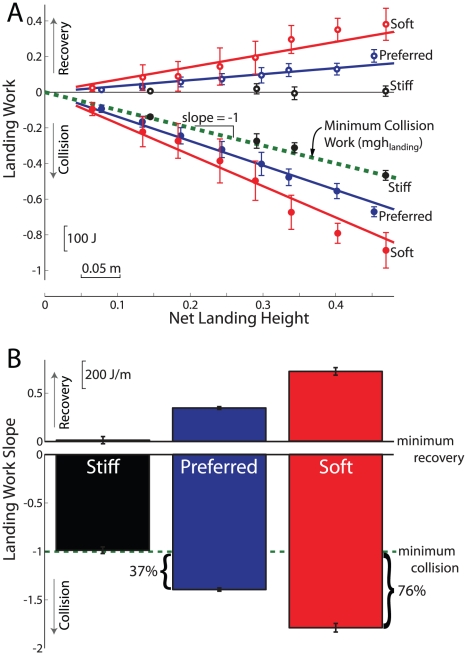
Total landing work: plotted (A) as a function of net landing height and (B) relative to minimum possible work for each landing phase and strategy. The theoretically minimum amount of landing work possible was defined as the change in potential energy from peak aerial height to standing rest (dashed line). Stiff landings achieved very close to this theoretical minimum. During Preferred landings, subjects performed 37% more Collision work than the minimum, necessitating additional positive Recovery work. Soft landings were even more extreme, with subjects performing about 76% more negative work than necessary. All relative work amounts were significantly different from each other (*P*<0.001).

Work summary measures were integrated from the power estimates over various jump phases. We divided each trial into phases – Counter-Movement, Push-Off, Aerial, Collision, Recovery – defined by separate regions of positive and negative COM work (Fig. 1). The Collision and Recovery phases together account for the work of landing. Power and work measures were non-dimensionalized before regression analysis to account for size differences between subjects, using body mass (*M*), leg length (*L*) and gravitational acceleration (*g*) as base units. All results are presented in dimensionless units. Mean power and work normalization constants were *Mg* ^3/2^ *L* ^1/2^ = 2302 W and *MgL* = 712 J, respectively. We computed work measures for each trial individually, and then performed linear regressions on Total and Soft Tissue work with respect to net landing height. Student's t-tests were used to compare regression coefficients and determine statistical significance at a level of *P*<0.001. We performed fits to *W* = *Bh*, where *W* is work, *h* is net landing height, and *B* is the slope coefficient, with an assumed offset of zero. We defined the proportion of work done passively during each phase of the jump as the ratio of the Soft Tissue work coefficient (*B*_SoftT_) divided by the Total work coefficient (*B*_Tot_).

Finally, we performed methodological control tests to validate the novel Soft Tissue work estimates. We asked each subject to perform 10 squatting trials, which involved squatting down slowly, then returning to resting posture. This yielded measurements of joint rotations similar to the jumping trials, but without the aerial phase or jarring landing collision.

## Results

Mechanical work varied consistently with net landing height and landing strategy. We generally observed the Preferred landings to involve more negative Collision work overall than the minimum theoretically possible. And Soft landings tended to involve more work than Preferred. When negative landing work was performed in excess of the minimum possible, extra positive work followed in order to return to standing rest. The Total Collision work was distributed between a combination of Joint and Soft Tissue contributions, with the amount and distribution also varying systematically with landing height and strategy. The contribution of Soft Tissue work to the Collision was highest at low landing heights, and approached an approximately constant percentage for higher jumps. During Soft landings we observed more work overall, especially from Joint contributions, and during Stiff landings we observed less work, but with increased Soft Tissue contributions.

During Preferred landings, subjects performed more Total negative work than necessary. At the greatest net landing height of about 42 cm, subjects performed about −477 J of negative Collision work, and then 145 J of positive Recovery work. The theoretical minimum Collision would be about −319 J from the potential energy of body weight (759.4 N) at that height, followed by 0 J of Recovery. The amount of negative Collision work changed approximately linearly with net landing height (Fig. 2A). This work is described by the work coefficient *B* (total landing work per unit landing height), which in dimensionless units may be interpreted as a relative amount of Collision work compared to the theoretical minimum. A work coefficient of −1 therefore corresponds to that minimum. In Preferred landings, the relative Collision work was −1.37±0.01 (mean ±95% confidence interval) from a linear fit (*R* ^2^ = 0.96), meaning that subjects performed 37% more negative work than minimally necessary (*P*<0.001; Fig. 2B). This was then followed by a similar amount of positive Recovery work to return to standing rest, with slope 0.339±0.007 (*R* ^2^ = 0.76), which was significantly different from zero.

The amount of work could also be modulated by different landing strategies (Fig. 2). In the Soft landing strategy, subjects performed 76% more Collision work than necessary (slope = −1.76±0.02; *R* ^2^ = 0.84), followed by a similar amount of positive Recovery work (0.707±0.020; *R* ^2^ = 0.59). The single subject who performed the Stiff landing was able to achieve Collision work magnitudes within 4% of the theoretical minimum, −0.969±0.017 (−735.7±13.1 J/m; *R* ^2^ = 0.97), followed by an insignificant amount of positive work after landing, 0.013±0.017 (*P* = 0.37; *R* ^2^ = 0.01). The Collision work of Soft and Preferred landings were significantly different from each other and from the theoretical minimum. For example, for the highest net landing heights (approximately 42–44 cm), the Soft landing Collision work was about −630 J, compared to −480 J for Preferred and −330 J for Stiff landings.

**Figure 3:**
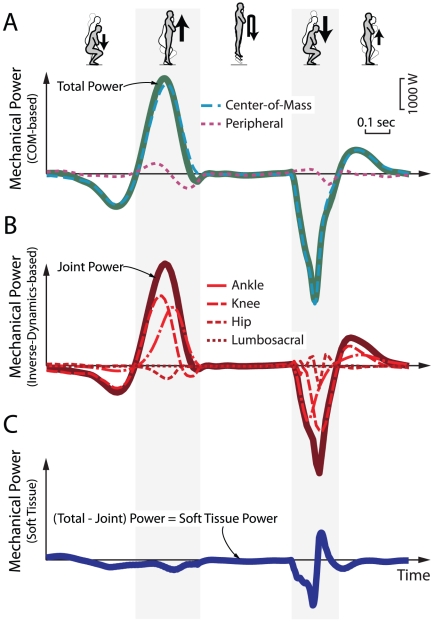
Mechanical power estimates. (A) Total power was estimated as the sum of center-of-mass (COM) power (due to motion of the COM) and Peripheral power (due to motion relative to the COM). (B) Joint power represents net contributions from muscle-tendon acting about the joints (ankle, knee, hip, lumbosacral), based on standard inverse dynamics. (C) Soft Tissue power is defined as the Total power minus the Joint power (see Text S1 for more details on calculations). Soft Tissue power was close to zero in most phases of the jump, except during Collision (immediately after touchdown), when it exhibited regions of negative, then positive power.

**Figure 4:**
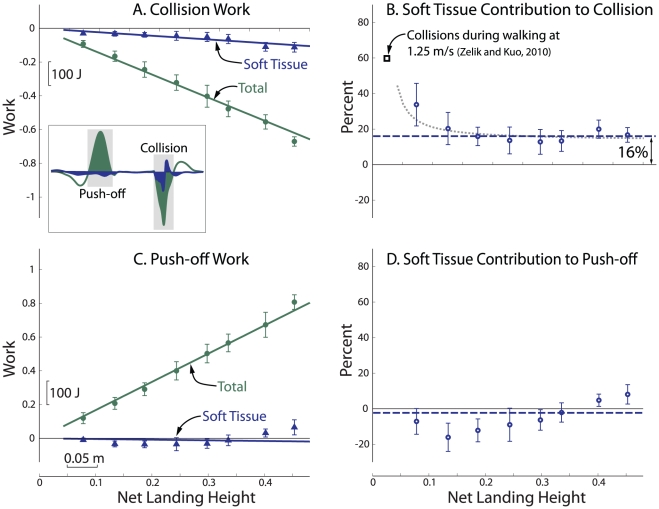
Collision and Push-off work, and contributions from passive soft tissues to Total work during Preferred landing. Total and Soft Tissue work are plotted as a function of net landing height for (A) Collision and (C) Push-off (*N* = 8). Passive contributions were computed as the ratio of linear regression slopes (*B*_SoftT_/*B*_Tot_) for (B) Collision and (D) Push-off, yielding asymptotic proportions of 16% and −2%, respectively (shown as dashed lines). Soft Tissues therefore contributed substantially to Collision, but not to Push-off. For low net landing heights, the Soft Tissue contribution to Collision appeared to be greater than the asymptote (deviation shown as gray dotted line). That deviation appears consistent with heelstrike Collisions during walking, which are similar in magnitude to landings of about 3 cm and have Soft Tissue contributions of about 60%, as estimated previously [12].

We observed indications of substantial Soft Tissue work, specifically during Collision (Figs. 3, 4). Total Mechanical power exhibited a large positive region of Push-off before take-off, followed by a large negative region of Collision immediately after touchdown. Summed Joint power followed a similar pattern, but with a lower magnitude of negative work during Collision, indicating contributions from Soft Tissue work (Fig. 3). Representative Joint powers are shown in (Figs. S2, S3, S4), as are two other independent indicators of Soft Tissue work (Fig. S1), further demonstrating that Joint work estimates fails to account for the Total work performed during landing.

Joint and Soft Tissue Collision work both increased with net landing height (Fig. 4). For Preferred landings, the Soft Tissue work of Collision was about −79.8 J for net landing heights of 42 cm (Fig. 4A). Soft Tissue Collision work exhibited an approximately linear relationship with net landing height, with work coefficient −0.215±0.005 (*R* ^2^ = 0.50). Soft Tissue work was therefore about 16% of Total Collision work, a proportion approached at greater landing heights (Fig. 4B). But the ratio was generally higher for low landing heights, for example 34% at 7.3 cm, with some subjects exhibiting percentages as high as 50–70% on individual trials.

**Figure 5:**
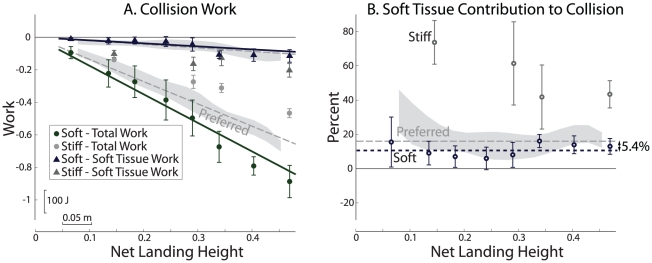
Collision work, contributions from passive soft tissues during Soft and Stiff landing. (A) Soft landings exhibited a significant increase in magnitude of Total work and a significant decrease in magnitude of Soft Tissue work during Collision, causing (B) a reduction in Soft Tissue contributions from the Preferred 16% to 10.6%. Stiff landings had the opposite effect, reducing Total Collision work and increasing Soft Tissue Collision work, with the overall effect of substantially increasing the proportion of Collision performed passively.

Subjects were able to modulate the amount and distribution of work during landing Collisions as a function of landing strategy. During Soft landings, subjects performed more Collision work through Joint rotations, and thus less through Soft Tissue than during Preferred landings (Fig. 5B, 10.6% vs. 16.0%). This difference was primarily due to a significant increase in the magnitude of Total Collision work, and to a lesser extent to a significant decrease in the magnitude of Soft Tissue work, −0.186±0.007 vs. −0.220±0.005 (Soft vs. Preferred, Fig. 5A), with the relative differences indicating an increase in Joint work. The opposite occurred with the single subject's data for Stiff landings, where Soft Tissue work constituted as much as 60–80% of the Total Collision work, a percentage that decreased with net landing height.

**Figure 6:**
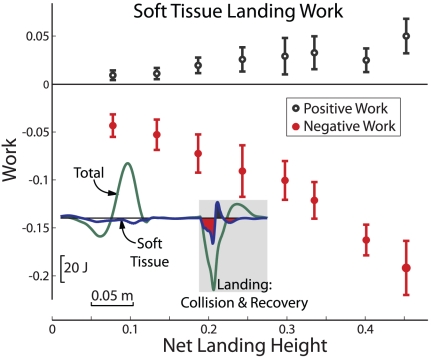
Soft Tissue work after touchdown in Preferred landing. Positive Soft Tissue work immediately following the negative Collision work suggests an elastic rebound of passive tissues. For all landing conditions, the magnitude of positive Soft Tissue work after landing was about 20–30% of the negative work.

Following the initial region of negative Soft Tissue work after touchdown, we observed a region of positive Soft Tissue work (Fig. 6). This was evident for all landing conditions and typically occurred during the Collision phase (when the body overall was performing negative work). In some cases it also continued into Recovery (when overall positive work was performed). The positive Soft Tissue work increased with Collision magnitude, and was typically about 20–30% of the magnitude of the negative Soft Tissue work (Fig. 6).

As a methodological test of the quantification of Soft Tissue work, we also examined other phases of the jump and the control squatting trials. Push-off and Counter-Movement phases would be expected to be less impulsive and more active, and therefore should be well explained by the measured Joint work. Indeed, Soft Tissue work, accounted for only −2.3% of the Total work during Push-off (slopes −0.039±0.010 and 1.674±0.009 for Soft Tissue and Total, respectively). And Soft Tissue work (slope −0.034±0.003) accounted for less than 6% of the Total Counter-Movement work (slope −0.588±0.008) across net landing. Similarly, during the squatting trials, Joint work was within 6% of Total work. Positive work amounts were 0.249±0.063 (Joint) vs. 0.263±0.066 (Total), mean ± standard deviation, and negative work amounts were −0.254±0.064 (Joint) vs. −0.270±0.068 (Total). This was equivalent to averages of 177 J of Total positive work, 187.1 J of positive Joint work, −180.7 J of Total negative work, and −192.1 J of negative Joint work. We also tested for internal consistency of each of these work measures by summing positive and negative work over the full squatting trial. As expected, both measures summed close to zero, 0.005±0.007 and 0.007±0.005 for Total and Joint work, respectively (equivalent to 3.7 J and 5.0 J).

## Discussion

Although humans seem to value economy of movement [1]–[7], they might prefer to perform extra muscle work to avoid other subjective costs, such as excessive soft tissue deformations during large Collisions that could cause pain or risk of injury. We found that subjects normally preferred a landing strategy that involved performing 37% more negative Collision work than necessary (Fig. 2). This extra work then required an equal amount of positive Recovery work to return to standing rest. This consequence was highlighted by Soft landings that entailed 76% more negative work than necessary, followed by a similar amount of positive Recovery work. The preferred amount of extra work performed, and its associated energetic cost, may reflect how the subjects value the trade-off between economy and subjective costs such as pain. Humans appear to be willing to consistently exchange an extra amount of mechanical work to avoid costs associated with landing too stiffly. We propose that quantification of this work may thus serve as an indicator of a person's subjective valuation of that preference.

Subjects preferred a landing strategy that involved a combination of active and passive Collision work. The proportion of work performed passively by Soft Tissues was typically 16% for moderate to high jumps (Fig. 4). This amount may seem modest, but is actually comparable to the contribution from any individual joint during landing [8]–[11]. Thus, the error from not accounting for Soft Tissue work is about the same as that from failing to include one of the leg joints. The proportion may even be greater for relatively small Collisions, where in some cases Soft Tissue contributions exceeded the combined contributions from all the joints. Landing Collisions from the lowest heights were similar in magnitude to those observed in the Collision following heelstrike in walking, and the relative passive contributions were in relatively good agreement with previous estimates of 60% for walking [12], [13]. The preference to perform work more passively during small collisions and more actively during larger Collisions is consistent with the proposed trade-off between economy and other costs, since we expect humans would be willing to exert more muscle effort to avoid costs associated with larger and potentially more damaging Collisions.

Subjects were able to modulate the distribution of work between active and passive tissues. When instructed to land softly, subjects performed a higher percentage of the Collision work actively, and when instructed to land stiff-legged the subject performed more passively, consistent with some preliminary quantifications [14]. Soft landing trials were subjectively reported to be more fatiguing than Preferred, while the subject who demonstrated Stiff landings reported substantial discomfort in his knees and lower back. During Soft landings, subjects performed on average 89.4% of the Collision work actively. We suspect that the proportion could be increased in actual practice, when humans may also use their arms and other joints to perform negative work, or perform more complicated land-and-roll maneuvers as in martial arts. However, since more than 80% of the body is comprised of “soft” (i.e., non-skeletal) tissues [28], there may be some practical limit to the maximum percentage of Collision work that can be done by active joint rotations when landing from a given height. Strength and flexibility may also be factors limiting an individual's ability to absorb Collision over an extended duration (Fig. S5), as previously observed in comparing athletes vs. sedentary individuals in a drop landing task [29]. Alternatively, the Stiff landing condition demonstrated that humans are capable of performing most of the Collision work passively. In fact, landings could potentially be performed completely passively if a subject were to simply fall limply onto the ground, although that could be painful and would also require more positive Recovery work to return to standing rest. Of course, there are practical limitations to testing this empirically, which is why we did not test the hypothetical example of walking vs. falling down stairs. It nevertheless appears that humans can choose a wide variety of landing strategies, of which their preference is quite consistent and involves more work than necessary.

Soft tissues also appear to perform some positive work during a damped-elastic rebound after landing. We observed fluctuations in Soft Tissue power after touchdown, initially negative, then followed by a region of positive power. While passive tissues can only perform net negative work, elasticity of these tissues could allow them to store and return some energy, for example in the bouncing of viscera [12], [30], [31]. Independent of the landing condition, the energy returned by this damped-elastic rebound appeared to be about 20–30% (Fig. 6). Similar evidence for a damped rebound of soft tissues has previously been observed in walking, with energy returns of about 10% at 1.25 m/s [12]. These are, however, very rough estimates because soft tissues could elastically rebound on a variety of time scales. The Soft Tissue measure only captures the net power resulting from the simultaneous deformations of many distributed soft tissues in the body. Nevertheless, this passive elastic rebound could affect the energetic economy or preferred frequency of cyclic movements, such as walking, running or hopping.

Our experimental estimates of mechanical work are subject to a number of limitations. We assumed that work done about the ankle, knee, hip and lumbosacral joints accounted for most of the active work of jump landing. It is also possible that substantial work is performed about other, unmodeled joints, or that some joint work is performed passively by series elastic elements, or that our mechanical work estimates are simply inaccurate. However, our methodological tests suggest that the Joint estimates are reasonably accurate since they yielded approximately zero net work as required for full-cycle squatting trials, and agreed well with Total work during the non-Collision phases of the jump cycle. We also found that the indirect estimate of Soft Tissue work during landing was supported by two other independent findings (Fig. S1), which both suggested that the magnitude of negative work done by passive tissues increased with net landing height. One indicator was the imbalance between positive and negative Joint work over the entire jumping and landing task, which should sum to zero. The other was that the Joint work magnitude after touchdown was less than the potential energy change from peak aerial height. This agreed with the observed temporal aspects of Soft Tissue work, specifically that Joint work measures failed to capture substantial negative work after touchdown, work we attribute to passive tissue deformations. Collectively, these separate yet corroborating indicators suggest that passive tissues do indeed perform substantial work during jump landings.

A different limitation is that our estimates do not indicate where in the body the passive work is performed. Other techniques, perhaps using imaging or direct strain measurement (e.g., [32]–[34]), may provide more detail regarding passive dissipation and damped-elastic rebound. Another limitation is that unlike COM work, the Peripheral work estimates only capture “rigid” work, and not that due to passive tissue within a segment relative to that segment's COM. Errors from that assumption might be expected to cause an imbalance in Total work over a full jump cycle; however, Total work summed close to zero (Fig. S1), providing some support for this assumption. A fourth limitation is related to the challenge of selecting filter parameters for force and motion data, which have been shown to affect joint moment impact peaks, typically within the first 30 ms of touchdown [35]. Therefore, we checked if our conclusions were sensitive to these parameters. Informal experimentation with different filter cut-off frequencies had little effect on the magnitude of negative Soft Tissue work estimates during Collision, although higher motion cut-off frequencies did tend to increase the subsequent burst of positive work, which we have speculated might represent a damped elastic rebound of passive tissues. Therefore, the estimated 20–30% efficiency of the damped passive rebound may actually be an underestimate. Finally, in this study we only consider the work performed by muscles, and not other contributors to energy expenditure such as force or rate of force production. Literature suggests that the muscle work is more metabolically costly than producing the same force isometrically [36], but there may nonetheless also be costs associated with producing force. These and other costs are best addressed through separate experiments designed to reveal their nature (e.g., [37], [38]).

We have alluded to subjective costs that might influence how humans prefer to land from a jump. Subject feedback suggested that pain-like factors might be one of the prevailing costs associated with preferred landing strategy, but with our current methods we have no way to extract the dominant factors from the pool of possible subjective costs. A variety of other subjective factors such as balance [39], safety, gracefulness, societal expectations, and even fun might also be relevant for motor tasks in general. If it were possible to hold all these other factors constant, previous findings [1]–[7] suggest that humans would choose to minimize active muscle work, and thus metabolic cost. Minimizing energy expenditure has been implicated as one factor influencing continuous, cyclic motions, such as walking [1]–[7], and while its relative importance may be diminished in some discrete movements, such as jump landings, it is still expected to be an influential factor since continuous motions could simply be thought of as a sequence of discrete actions.

We propose that mechanical work or energy may serve as a common currency for evaluating trade-offs that are otherwise difficult to quantify. If work were the only factor influencing preference, we would expect it to be minimized, but the degree to which it is not minimized in certain tasks may provide insight into the relative importance of other factors. This idea of work as a common currency seems to be consistent with other landing studies, which indicate that people choose to perform more work when landing on stiff surfaces than on compliant, and presumably more comfortable, surfaces [40]–[42]. We actually would prefer metabolic energy as a currency for its greater physiological relevance, but it is less amenable to measurement in discrete tasks such as considered here. Unlike force or kinematic measures, work and energy are both objective, scalar variables, regardless of how many and which joints are involved in a task. Work is part of most motor tasks, making it an appropriate currency for evaluating subjective factors that might not be as common to movement strategies in general. While this study provides an initial impetus for the use of mechanical work as a means of indirectly quantifying subject costs, further research is needed to validate work as a common currency. Future studies might explore, for instance, how the partitioning of work changes with fatigue or discomfort. Ultimately, by manipulating subjective factors that influence preference, it may be possible to alter the distribution of work and economy of movement. For instance, by changing characteristics of biomechanical aids (e.g., shoes), we may be able to alter the importance of other subjective factors (e.g., comfort) such that humans prefer to perform a task more or less economically. Mechanical work would serve as an indirect measure of the relative weighting of these other factors, and could therefore be a useful metric for selecting components or properties for such aids.

The preferred strategy for a motor task is presumably a balance between competing trade-offs. Some may be quantifiable like work, and others may be difficult to define, let alone quantify, like pain. Biomechanical measures usually focus on active motions performed by muscle, but it appears that passive deformation of soft tissues may also be important. The work they perform can affect what is needed from active muscle, as well as the pain that is felt by both active and passive tissues. It might seem obvious why humans prefer to walk rather than fall down a flight of stairs. But less obvious is that the work performed by doing so may also be an indirect valuation of the relative costs of falling or other alternatives.

## Supporting Information

Text S1 **Appendix: Detail regarding calculation of work measures** [**43]**, [44****] **.**
(PDF)

Figure S1 **Additional indicators of Soft Tissue work.** (A) Net work done over the entire jump-landing cycle should sum to zero. This was observed for Total mechanical work, but not for summed Joint work. (B) Net work done during landing minus the change in potential energy during aerial descent should also sum to zero. Similarly, Total mechanical work sums to zero, as expected, but Joint work does not. Both independent methods indicate that Joint work estimates fail to capture substantial work, increasing with net landing height.
(EPS)

Figure S2 **Joint angles, moments and powers for a range of net landing heights (representative trials from a single subject).** Data are only plotted for periods of ground contact when the primary work was performed. Plots are aligned at times of take-off and touchdown, so that differing time durations of aerial phases are not shown; a typical duration of 200 ms is shown for reference.
(EPS)

Figure S3 **Joint angles, moments and powers for Preferred, Soft and Stiff landings conditions with net landing height of 0.12 m (representative trials from a single subject).** Data are only plotted for periods of ground contact when the primary work was performed. Plots are aligned at times of take-off and touchdown, so that differing time durations of aerial phases are not shown; a typical duration of 200 ms is shown for reference.
(EPS)

Figure S4 **Joint angles, moments and powers for Preferred, Soft and Stiff landings conditions with net landing height of 0.46 m (representative trials from a single subject).** Data are only plotted for periods of ground contact when the primary work was performed. Plots are aligned at times of take-off and touchdown, so that differing time durations of aerial phases are not shown; a typical duration of 200 ms is shown for reference.
(EPS)

Figure S5 **Time duration of Collision phase.** Soft landings, which were associated with increased active muscle work, exhibited extended Collision durations (*P*<0.001). Stiff landings, which were associated with increased passive soft tissue work, exhibited shortened Collision durations.
(EPS)
