# Supplementary material for: Mechanical Work as an Indirect Measure of Subjective Costs Influencing Human Movement
Source: PLoS One. 2012 Feb 24;7(2):e31143. doi: 10.1371/journal.pone.0031143 (PMC3286468; doi:10.1371/journal.pone.0031143)
Supplement: Text S1 — Appendix: Detail regarding calculation of work measures [43] , [44] . (PDF) [file pone.0031143.s001.pdf]

Text S1: Appendix

Detail regarding calculation of work measures is given here. We defined Soft Tissue power (P_{SoftT}) as the difference between Total mechanical power (P_{Tot}) of the body and inverse dynamics estimated Joint power (P_{Joint}),

$$P_{\mathrm{SoftT}} = P_{\mathrm{Tot}} - P_{\mathrm{Joint}}$$

where Total power was defined as the sum of power due to motion of the COM (P_{COM}) plus power due to motion relative to the COM ($P_{\mathrm{Peripheral}}$), according to König's Theorem [43]:

$$P_{\mathrm{Tot}} = P_{\mathrm{COM}} + P_{\mathrm{Peripheral}}$$

COM power (or work rate) was defined as the dot produce of the ground reaction forces (F_{i}) with the velocity of the COM (v_{COM}). Forces were measured under each foot *via* force plates. The velocity of the COM was integrated from the acceleration of the COM (a_{COM}, equal to the sum of the forces divided by the subject mass, m), assuming zero average velocity over each jumping trial.

$$P_{\mathrm{COM}} = \sum_{legs} F_i \cdot v_{\mathrm{COM}}$$

$$v_{\mathrm{COM}} = \int a_{\mathrm{COM}} dt = \int \frac{\sum F_i}{m} dt$$

Peripheral power was estimated as the time derivative of the translational kinetic energy of each segment with respect to the COM plus the and derivative of segmental rotational kinetic energy. Segmental velocities (v_{s}) and angular velocities (w_{s}) were based on filtered marker motions, assuming rigid-bodies (with a minimum of four markers on each segment). Inertial properties (I_{s}, m_{s}) of each segment (foot, shank, thigh, pelvis, head-arms-trunk) were estimated based on anthropomorphic regression tables [44].

$$P_{Peripheral} = \sum_{segments} \frac{d}{dt}\left(\frac{1}{2} m_s (v_s - v_{COM})^2 + \frac{1}{2} I_s \omega_s^2\right)$$

This estimate fails to capture some of the motion of tissue within a segment relative to that segment's COM. This contrasts with COM work rate, which does include the contributions of soft tissues to overall COM motion. Our measure of Peripheral power therefore assumes that most of the work performed relative to the COM is quantified by the motion of rigid body

segments. Methodological tests detailed in the discussion suggest that the error from this assumption is not large.

Standard 3-D inverse dynamics were used to estimate power (the dot product between joint moment, M_j, and joint angular velocity, w_j) about the ankle, knee, hip and lumbosacral joints, then summed to obtain net Joint power.

$$P_{Joint} = \sum_{joints} M_j \cdot \omega_j$$
